# Multimodal foundation models in colorectal cancer: from prediction to trustworthy clinical insight

**DOI:** 10.1093/bib/bbag179

**Published:** 2026-04-27

**Authors:** Danial Gharaie Amirabadi, Adib Miraki Feriz, Hossein Safarpour

**Affiliations:** Student Research Committee, Birjand University of Medical Sciences, PO Box 9717853577, Birjand, Ghaffari Street, South Khorasan Province, Iran; Cellular and Molecular Research Center, Birjand University of Medical Sciences, PO Box 9717853577, Birjand, Ghaffari Street, South Khorasan Province, Iran; Cellular and Molecular Research Center, Birjand University of Medical Sciences, PO Box 9717853577, Birjand, Ghaffari Street, South Khorasan Province, Iran

**Keywords:** colorectal cancer, multimodal foundation models, functional space navigation, lab-in-the-loop, trustworthy AI, precision oncology

## Abstract

Colorectal cancer (CRC) is characterized by profound, multi-layered heterogeneity that limits the precision of conventional single-modality clinical tools. The emergence of multimodal foundation models (MFMs) represents a conceptual paradigm shift, moving beyond static biomarkers to capture the dynamic and evolving nature of CRC. MFMs integrate histopathology, radiology, multi-omics data (including the critical regulatory layer of epigenomics), and clinical variables into shared high-dimensional representational spaces. This integration enables improved prognostication, refined molecular subtyping, and *in silico* simulation of therapeutic perturbations within the tumor’s functional landscape, thereby supporting rational and model-driven drug development. In this review, we synthesize the rapidly expanding body of CRC-specific MFM research and critically examine the unresolved challenges that currently limit clinical translation. We place particular emphasis on the transition from correlation to causal inference, the establishment of cross-population generalizability, and the resolution of key issues related to trustworthiness and clinical interpretability. Finally, we propose an actionable roadmap outlining regulatory, data governance, and translational requirements, including the lab-in-the-loop paradigm, necessary to position MFMs as a robust and equitable framework in clinical oncology.

## The clinical imperative: managing the heterogeneity of colorectal cancer

Colorectal cancer (CRC) is the third most commonly diagnosed malignancy and the second leading cause of cancer-related mortality worldwide [1]. Despite substantial advances in molecular characterization, therapeutic decision-making remains hindered by pronounced heterogeneity at multiple levels. Inter-patient heterogeneity results in markedly different disease trajectories and treatment responses among patients with identical TNM stages, while intra-patient heterogeneity encompasses both spatial variation (e.g. tumor core versus invasive front) and temporal evolution (e.g. pre- versus post-treatment biological states) [2].

Current clinical decision-making relies on a limited set of genetic biomarkers, including RAS (KRAS, NRAS), BRAF mutations, as well as mismatch repair deficiency (dMMR) or high microsatellite instability (MSI-H) [3, 4]. Although these biomarkers are clinically actionable, they apply to only a subset of patients and fail to capture the full biological complexity underlying CRC heterogeneity [5].

Comprehensive molecular profiling, particularly through RNA sequencing, has enabled broader biomarker discovery and the development of consensus molecular subtypes (CMS) [6, 7]. However, these approaches remain constrained by their static snapshots as they capture tumor state at a single time point and provide limited insight into dynamic processes such as therapeutic adaptation, metastatic dissemination, and microenvironmental reprogramming [8].

This limitation have driven the development of computational ‘virtual tumor’ models, designed as high-fidelity digital representations of patient-specific tumors capable of simulating disease evolution and treatment response. Pioneering initiatives, such as the Center for the Development of a Virtual Tumor (CViT) platform at Massachusetts General Hospital, integrate multimodal data to generate predictive simulations of tumor behavior under therapeutic perturbations [9, 10]. While these frameworks underscore the clinical need for dynamic, patient-specific modeling, they remain constrained by fragmented data integration and lack of generalizability.

The advent of multimodal foundation models (MFMs) represents a paradigm shift beyond traditional static biomarker frameworks. Trained in a self-supervised manner on large and diverse biological and clinical datasets, MFMs capture fundamental principles governing molecular and cellular systems [11, 12]. Generative MFMs, in particular, extend beyond correlation-based prediction by enabling simulation of tumor adaptation, resistance trajectories, and therapeutic perturbations within high-dimensional functional spaces [13].

Crucially, MFMs unify disparate data modalities, including histopathology, radiology, genomics, transcriptomics, proteomics, metabolomics, spatial omics, epigenomics, and electronic health records (EHRs), into shared representational frameworks [14, 15]. This integration enables CRC to be modeled not as collection of isolated data layers, but as an interconnected, evolving system. Early evidence supports this potential. Hu *et al.* (2025) demonstrated that a pan-cancer MFM integrating histopathology and genomics data improved prognostic accuracy across 18 tumor types, highlighting the presence of shared biological principles across malignancies [16].

By capturing both inter-patient variability (e.g. prognostic stratification) and intra-patient dynamics (e.g. spatial niche interactions and clonal evolution), MFMs pave the way for a future in which treatment decisions are informed by projected disease trajectories rather than retrospective assessments.

This review synthesizes the accelerating landscape of CRC-specific MFMs, critically evaluates unresolved challenges in causal inference, generalizability, and equity, and proposes a trustworthy translational roadmap, positioning CRC as a proving ground for MFMs in oncology.

From a practical clinical perspective, the most translation-ready MFMs rely on data that are already routinely collected within standard-of-care workflows, including hematoxylin and eosin (H&E)-stained whole-slide images (WSIs), CT, and MRI radiology scans, basic EHR variables (age, disease stage, tumor site, treatment history), and targeted gene-panel sequencing or immunohistochemical surrogates such as mismatch repair protein staining. Fully multimodal models that also ingest bulk or single-cell transcriptomics, epigenomics, or proteomics currently remain research-only because these assays are not universally performed; however, ongoing work aims to impute or predict these high-dimensional layers from routine H&E and clinical data alone, potentially eliminating the need for additional patient sampling.

To ensure relevance and coherence, models discussed in this review were selected according to predefined criteria. We prioritized architectures that (i) integrate two or more biomedical data modalities or provide a feasible pathway toward multimodal extension, (ii) demonstrate transferable utility across cancer types or large-scale biomedical tasks, (iii) report clinically meaningful endpoints (e.g. prognosis, MSI, treatment response), with external validation when available, and (iv) function as reusable or foundational backbones rather than single-purpose models. Architectural diversity was deliberately maintained, while models lacking transparency or clear translational relevance were deprioritized.

## The technological revolution: the rise of foundation models in colorectal cancer

**Figure 1:**
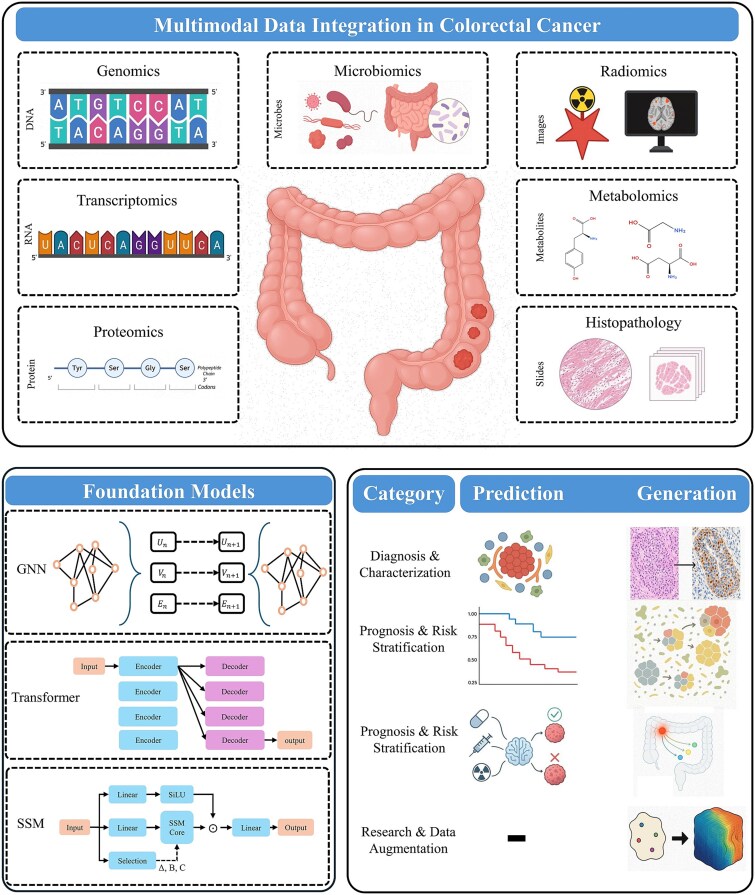
Conceptual overview of multimodal foundation models in colorectal cancer (CRC). Diagram illustrating how multimodal foundation models integrate genomic, transcriptomic, imaging, and clinical data to analyze colorectal cancer biology and generate predictive and mechanistic insights.

Foundation models (FMs) initially transformed natural language processing through large-scale self-supervised pretraining, enabling robust generalization across diverse downstream tasks [11]. This paradigm has since permeated biomedicine, driven by the exponential expansion of genomic, transcriptomic, imaging, and clinical datasets. In CRC, a disease defined by the complex interplay of somatic mutations, tumor microenvironment (TME) dynamics, and host–microbiota interactions, FMs provide a unifying framework not only for predictive modeling but also for hypothesis generation and mechanistic simulation (Fig. 1).

FMs are broadly classified as discriminative or generative. Discriminative models, typically encoder-based (e.g. BioBERT [17], DNABERT [18], and ProteinBERT [19]), excel at extracting high-dimensional embeddings for downstream classification tasks such as MSI status prediction or biomarker identification. In contrast, generative architectures which are generally decoder-based (e.g. ProtGPT2 [20], ProGen [21], and ESM [22, 23]) synthesize biological sequences, impute missing data, or simulate biological perturbations, enabling applications in protein design and drug response forecasting. In CRC, this distinction translates into complementary strengths: discriminative FMs facilitate patient stratification, whereas generative FMs provide an unprecedented capacity to model tumor adaptation under therapeutic pressure, a capability largely absent from conventional biomarker-driven frameworks [13].

### The architectural pillars of modern foundation models

#### Transformers and self-attention

The transformer architecture, introduced in 2017, replaced recurrence and convolutional operations with self-attention, enabling each token, whether a DNA k-mer, image patch, or gene expression value, to compute weighted relationships with all other tokens in the sequence [24]. This mechanism is particularly powerful in CRC, where long-range dependencies regulate both genomic control elements (e.g. distal enhancers) and tissue-level architecture (e.g. tumor budding patterns associated with metastasis risk).

Vision Transformers (ViTs) have established new performance benchmarks in computational pathology [25]. Wagner *et al.* (2023) analyzed thousands of WSI patches to predict MSI status, RAS/BRAF mutations, and CMS, achieving performance superior to state-of-the-art convolutional neural networks (CNNs) [26]. However, the necessity of billion-parameter ViTs remains a subject of debate. Distilled model variants achieve near-equivalent MSI prediction performance on The Cancer Genome Atlas Colon Adenocarcinoma (TCGA-COAD) dataset while requiring <10% of the computational cost [27], raising critical questions regarding whether large-scale pathology FMs are over-engineered for clinically focused endpoints. Hierarchical architectures, such as Swin Transformers, partially mitigate quadratic computational scaling inherent to self-attention [28]. Nevertheless, domain shift arising from variability in staining protocols, scanners, and institutional practices remains a persistent barrier to real-world clinical deployment.

#### Graph neural networks

Unlike transformers, which primarily operate on sequential or grid-structured data, graph neural networks (GNNs) are explicitly designed to model relational topologies. Through iterative message-passing mechanism, node embeddings are updated based on information exchanged with neighboring nodes, making GNNs well suited for representing gene regulatory networks, signaling pathways, and multicellular communities within the TME [29]. In CRC, models such as Nicheformer leverage GNN-based representations to identify functional neighborhoods of interacting immune and stromal cells, revealing immunosuppressive niches that correlate with resistance to immunotherapy [30]. However, the predominantly static nature of most GNN implementations represents a fundamental limitation. These models typically assume fixed graph topologies and therefore fail to capture clonal evolution, therapy-induced network rewiring, or temporal remodeling of the TME as processes that are central to CRC progression and therapeutic failure. Although residual connections and graph-rewiring strategies can partially alleviate over-smoothing [31, 32], temporal GNN architectures remain largely unexplored in oncology, representing a critical gap in the modeling dynamic disease trajectories.

#### State space models

State space models (SSMs), exemplified by architectures such as Mamba [33], offer a computationally efficient alternative attention-based models by representing sequences as latent dynamical systems with selective recurrence. This linear-time complexity makes them particularly well suited for ultra-long inputs, including whole-chromosome variant profiles or longitudinal clinical records.

In CRC, approaches such as dnaGrinder apply SSM-based architectures to interpret non-coding variants across megabase-scale genomic regions, enabling the identification of regulatory elements that are often missed by shorter-context models [34]. Nevertheless, model expressivity persists, as SSMs tend to underperform transformer-based architectures when modeling highly irregular dependencies, such as focal genomic amplifications or burst-like gene expression dynamics [35, 36]. Although hybrid architectures that combine SSMs with attention mechanisms are beginning to emerge, their application in CRC remains largely untested, leaving open the question of whether computational efficiency gains justify potential reductions in biological fidelity.

#### Hybrid and emerging architectures

The future of FMs increasingly lies in hybrid architectures that combine complementary strengths. CNN-transformer hybrids, such as Enformer, integrate local motif detection with long-range regulatory modeling to predict the functional effects of non-coding mutations [37].

Vision-language models align histopathology images with unstructured clinical reports, enabling multimodal reasoning across imaging data and EHR [38].

Spatially informed transformer, architectures such as Nicheformer, extend attention mechanisms to tissue-level organization, enabling the identification of multicellular niches that shape prognosis and therapeutic response [30].

Graph-transformer hybrid models further embed prior biological knowledge, such as protein–protein interaction networks and host-microbiome relationships, into representational latent spaces, yielding more mechanistically grounded model outputs [39].

Autoencoder-based architectures compress high-dimensional single-cell datasets, enabling the identification of rare, drug-resistant subclonal populations that are often obscured in bulk analyses [40, 41].

Despite these advances, no MFM has yet demonstrated robust zero-shot transfer across staining protocols, ethnic cohorts, or longitudinal timepoints without task-specific fine-tuning [42, 43]. The path forward likely lies not in ever-larger models, but rather in modular and interpretable fusion frameworks coupled with continual learning strategies capable of adapting to real-world clinical drift.

## Deconstructing the tumor: uni-modal foundation models in colorectal cancer

### The language of histology and radiology

Digital pathology, centered on the analysis of WSIs of H&E-stained tissue, remains a primary source of morphological information reflecting underlying molecular processes [44, 45]. Early deep learning (DL) applications relied predominantly on CNNs, which achieved notable success in tasks such as tumor grading and the prediction of molecular features including MSI status [46–48]. However, CNNs are inherently limited by their local receptive fields, restricting their ability to model long-range dependencies across large WSIs [49].

The introduction of transformer-based architectures, particularly ViTs, has addressed this limitation and established a new standard in computational pathology. By employing self-attention mechanisms, transformers can evaluate the relative importance of all image patches simultaneously, enabling a global understanding of tissue architecture that is essential for analyzing complex WSIs in CRC [50–53]. This capability is clinically relevant, as features extending beyond the primary tumor also influence prognosis. For example, imaging-derived features from metastatic sites such as texture patterns in liver metastases, can independently impact patient outcomes regardless of primary tumor stage.

In parallel, convolutional architectures continue to advance pathology FMs through innovative design strategies. A notable example is ROSIE, a ConvNext-based model capable of performing *in silico* multiplex immunofluorescence (mIF) by predicting biomarker expression directly from H&E-stained slides [54]. In a landmark study, Wagner *et al.* (2023) developed a transformer-based pipeline to predict key genomic and transcriptomic alterations in CRC from H&E slides across a multicenter cohort, demonstrating the potential of these approaches to function ‘digital molecular tests’ [26]. Beyond histopathology, attention-based models have also shown broad applicability in radiology, achieving near-expert-level accuracy in the challenging task of CRC tumor segmentation on computed tomography (CT) scans [55].

Despite these successes, substantial challenges remain. Domain shift arising from variability in slide preparation protocols, and scanning equipment across institutions can significantly degrade model performance, necessitating mitigation strategies such as stain normalization and training on large, diverse, multi-institutional datasets. Additionally, the sheer size of WSIs also presents a major computational challenge, requiring efficient patching strategies and hierarchical modeling approaches [56, 57].

An overview of FMs developed for histopathology and radiology applications in CRC is provided in Supplementary Table S1.

### The language of the genomic and transcriptomic

The genome serves as the fundamental blueprint of cancer, cataloging the landscape of somatic mutations and structural variants that underpin CRC classification [58, 59]. The ‘sequence-as-language’ paradigm, implemented through DNA language models like DNABERT, Enformer, and dnaGrinder [18, 34, 37], treats DNA sequences as linguistic constructs to learn the underlying grammar of gene regulation. This approach enables prediction of the functional impact of non-coding variants, extending beyond traditional variant calling.

While genomics provides the largely static blueprint, transcriptomics offers a dynamic snapshot of tumor activity, forming the basis of the landmark CMS framework [60]. More recently, AI-driven approaches have moved beyond established classifications. For example, the Gene Swin Transformer converts bulk transcriptomic data into synthetic image elements (SIEs) that can be processed by a vision transformer, enabling the identification of novel prognostic biomarkers such as *PEX10* [61].

Resolving tumor heterogeneity ultimately requires moving beyond the ‘blended’ perspective of bulk sequencing toward single-cell-level analysis. Single-cell foundation models (scFMs), such as Geneformer and scGPT [62, 63], are transformer-based architectures pretrained on millions of single cell profiles. This has led to the emergence of a broader ecosystem of models including scPRINT and scCello, which interrogate the TME at unprecedented resolution, enabling rapid cell-type annotation, inference of gene regulatory networks, and robust cross-species transfer learning [64–68].

Transcriptome FMs enable high-throughput, ontology-aware dissection of cell subpopulations, thereby refining our understanding of tumor heterogeneity and mechanisms of drug resistance. A key strength of these models lies in their ability to generate ‘digital’ surrogates for costly molecular assays, exemplified by the prediction of dMMR status directly from routine H&E slides [69].

The CRC TME represents a dynamic ecosystem in which spatial organization is critical to disease behavior. A new generation of FMs provides the tools to map this ecosystem through multi-stage analytical pipelines, including large-scale pre-trained models such as scFoundation [70] that function as universal cell annotators; spatial FMs (e.g. scGPT-spatial) that map cells back to their tissue context [71]; and transformer-based models like Nicheformer [30] that identify multicellular ‘niches’ as functional neighborhoods of interacting cells.

Representative genomic, transcriptomic, and spatial FMs relevant to CRC are detailed in Supplementary Tables S2 and S3.

### The language of function, regulation, and the ecosystem

#### Epigenomics: the regulatory layer

Epigenomic alterations, particularly DNA methylation and histone modifications, represent a crucial regulatory layer that governing CRC progression and therapeutic response; yet, they remain underrepresented in current MFMs [72]. DNA methylation is a well-studied epigenetic mark in which the addition of a methyl group to cytosine residues regulates gene expression, often resulting in the silencing of tumor suppressor genes [72]. This process is fundamental to the CpG island methylator phenotype (CIMP), a distinct molecular subgroup of CRC characterized by widespread promoter hypermethylation [73]. Emerging FMs are being developed to process this complex, high-dimensional data by treating DNA methylation profiles as sequences. Models such as MethylGPT are transformer-based architectures pretrained on large collections of human DNA methylation profiles to learn the complex, non-linear dependencies between distant CpG sites [74]. Similarly, the Cytosine-phosphate-Guanine Pretrained Transformer leverages a specialized transformer to impute missing methylation values and is applicable for various cancer prediction and classification tasks [75]. Integrating these epigenomic FMs with other omics and imaging modalities is critical, as it provides a mechanistic link between regulatory alterations and the observable tumor phenotype, such as immune evasion within the TME. The relative scarcity of large, clinically annotated epigenomic datasets therefore represents one of the most significant bottlenecks in building truly pan-omic multimodal FMs for CRC. Bridging this gap through epigenomic imputation from H&E images, spatial transcriptomics, or cell-free DNA methylome profiling constitutes an active and clinically critical research frontier.

#### Proteomics: decoding the molecular machinery

Mass spectrometry (MS)-based proteomics provides a direct readout of protein abundance, post-translational modifications (PTMs), and signaling activities that collectively define cancer cell function. Initiatives such as the ‘Clinical Proteomic Tumor Analysis Consortium (CPTAC)’ have generated deep proteogenomic maps of CRC, offering unparalleled opportunities to integrate proteomic signatures with genomic alterations [76, 77]. However, a central challenge lies in extracting functional meaning from observed changes in protein levels; as differential abundance alone often fails to capture downstream mechanistic consequences. This gap is increasingly addressed by ‘protein FMs’. Large-scale transformer architectures, such as ‘ESM’, trained on billions of protein sequences across the tree of life, learn intrinsic biophysical principles governing protein folding, enzymatic function, and interaction potential [22].

A recent innovation in this space is KRONOS, a FM for spatial proteomics [78]. KRONOS is trained on 47 million image patches across diverse tissues and leverages self-supervised learning to enable segmentation-free cell phenotyping, tissue region classification, and patient stratification. Its ability to generalize across platforms and sample types underscores the value of spatial proteomic FMs in CRC, where tumor-immune architecture and microenvironmental heterogeneity critically shape clinical outcomes. By coupling network-based reasoning with spatially resolved proteomic information, models such as KRONOS illustrate the next frontier of integrative CRC research. FMs for proteomics, protein structure, and molecular design are summarized in Supplementary Table S4.

#### Metabolomics: mapping the biochemical circuitry

Cancer cells, including those in CRC, undergo profound metabolic reprogramming to sustain anabolic growth, evade immune surveillance, and maintain redox balance, thereby producing distinctive metabolic signatures [79]. Interpreting these signatures requires computational methods capable of handling the graph-like topology inherent to metabolic pathways [80]. GNNs are particularly well suited to this task, as metabolites can be represented as nodes and enzymatic reactions as edges, allowing information to propagate through metabolic networks and enabling mechanistically interpretable links between genotype and metabolic phenotype [81].

In parallel, artificial intelligence is reshaping how metabolomic data are acquired, annotated, and interpreted. Early transformer-based models such as DreaMS, trained on millions of unlabeled MS/MS spectra, demonstrated that pretraining can infer fragmentation grammars and substantially improve metabolite identification [82]. Building on this foundation, large-scale FMs for metabolomics have recently emerged. PRISM, trained on over one billion spectra, applies masked spectral modeling to learn transferable chemical representations, resulting in marked improvements in chemical property prediction and spectral retrieval [83]. Similarly, LSM1-MS2 employs transformer-based self-supervision on tandem MS data, enabling robust metabolite annotation and *de novo* molecular generation, even in low-data regimes [84]. Beyond MS, recent proposals for NMR-based FMs extend self-supervised learning to nuclear magnetic resonance data, highlighting the potential of multimodal frameworks in metabolomics.

Concurrently, a growing ecosystem of task-specific transformer models is advancing metabolite annotation. The RT-Transformer integrates 1D-transformer layers with graph attention networks to enhance retention time prediction [85]. MassFormer, a graph transformer, accurately predicts MS2 spectra by learning atom-bond relationships and experimental conditions [86]. Models such as MS2Prop predict chemical properties directly from spectra [87], while IDSL_MINT [88] and MIST [89] generate molecular fingerprints for efficient library matching.

In lipidomics, LipiDetective achieves improved annotation of novel lipid species from MS2 spectra [90], and MS2Mol enables *de novo* structural prediction directly from spectral data without reliance on reference libraries [91]. CRC-relevant metabolomics and cheminformatics FMs are summarized in Supplementary Table S5.

#### Microbiomics: resolving the tumor-microbe axis

The gut microbiome is increasingly recognized as a pivotal determinant of CRC initiation, progression, and response to therapy [92]. However, advancing from correlative abundance profiles to causal mechanistic insights requires computational models that capture the community-level interactions within complex microbial consortia [93]. Graph transformer models, such as MICAH (MIcrobial Cancer-association Analysis using a Heterogeneous graph transformer), integrate abundance data, phylogenetic relationships, and metabolic capacities into a heterogeneous graph, enabling the identification of cancer-associated microbial communities rather than isolated taxa [92]. To facilitate biological translation, interpretability frameworks are also emerging. For example, IMPACT projects microbial abundance data into image-like representations and applies saliency mapping techniques from computer vision to highlight not only key taxa predictive of CRC risk but also their associated functional attributes [94]. This integrated interpretability is critical for generating biologically testable hypotheses and identifying potential therapeutic targets [95].

Beyond these task-specific architectures, microbiome research has also benefited from the adoption of large pretrained models, which demonstrated the value of transfer learning on high-dimensional microbial data [96]. Such approaches established the feasibility of capturing community-level representations at scale, thereby providing a bridge toward the development of dedicated FMs such as the Microbial General Model (MGM) [97]. The MGM treats each microbiome sample as a ‘document’ (a sequence of tokenized taxonomic or functional units), with individual taxa/OTUs/ASVs represented as ‘tokens’, enabling self-supervised learning and attention mechanisms to model co-occurrence structure and ecological interactions. Pretrained on >260 000 microbiome samples, MGM encodes rich contextual information and captures complex interspecies interactions, enabling downstream tasks such as disease classification, biomarker discovery, and microbiome-host interaction analysis relevant to CRC. Building on this framework, MGM 2.0 extends the paradigm toward ‘generative microbiome modeling’ [98]. By reframing microbial communities as language-like sequences, MGM 2.0 introduces generative capabilities including colonization prediction, disease-conditioned microbiome synthesis, and fecal microbiota transplantation donor selection. Although not strictly a FM, MGM 2.0 illustrates how transformer-inspired architectures can progress from representation learning to simulation, highlighting future opportunities for CRC research into therapeutic microbiome engineering. FMs for microbiome and metagenome analyses are shown in Supplementary Table S6.

Collectively, these functional omics layers and their associated AI models bridge the gap between genetic alterations and phenotypic manifestation. By capturing proteins, metabolites, and microbial networks as the executors and modulators of cancer biology, they provide a mechanistic framework that is essential for precision oncology.

Table 1 summarizes recent FMs and multi-omics models relevant to CRC, detailing their primary modality, key tasks, training scale, external validation, limitations, and clinical maturity.

**Table 1:** Representative FMs for CRC: CRC-related FMs with their corresponding tasks, applications, and limitations.

Model	Primary modality	Key tasks/CRC applications	Training scale/benchmarks	External validation	Key limitations	Clinical maturity
UNI (2024)	Histopathology (WSIs)	MSI prediction, outcome modeling, robust WSI embeddings	~100 K WSIs / ~100 M patches	Yes (multi-institution)	Domain shift persists; pathology-only	Preclinical (high maturity)
Virchow (2024)	Histopathology (gigapixel)	Pan-cancer detection incl. CRC, MSI prediction	1.5 M WSIs	Yes	Limited multimodality; interpretability challenges	Near-clinical
TITAN (2024)	Vision–language (WSI + reports)	Report-aware WSI retrieval, CRC biomarker prediction	335,645 WSIs with paired/synthetic text	Partial	Text variability reduces alignment	Preclinical
BiomedCLIP (2023)	Radiology + biomedical images + text	Radiology report grounding; CRC figure retrieval	15 M figure–caption pairs	Partial	General biomedical, not CRC-specific	Research
DNABERT-2 (2023)	DNA regulatory genomics	Promoters, enhancers, splice sites relevant to CRC	Multi-species genome corpora	Partial	DNA-only; limited tumor-specific fine-tuning	Research
Enformer (2021)	DNA → gene expression/epigenome	Noncoding variant effect for CRC risk loci	Long-range genomics (~200 kb receptive field)	Strong	Expensive inference; limited tumor context	Preclinical
scGPT / scGPT-spatial (2023–25)	Single-cell (+ spatial)	CRC TME cell states, spatial niches	33 M+ cells	Emerging	Very compute-heavy	Preclinical
Geneformer (2023–25)	Single-cell	Perturbation inference; CRC immune contexture	30 M single cells	Robust	Limited CRC-specific pretraining	Preclinical
CellFM (2025)	Single-cell (RetNet-based)	High-capacity cell-state embeddings for CRC	100 M cells	No	Very large model; limited public checkpoints	Early preclinical
ESM-2 (2022)	Protein sequences	Variant effect prediction, CRC protein function	Up to 15B params	Internal	No proteomics integration	High preclinical
KRONOS (2025)	Spatial proteomics	CRC cell phenotyping, TME architecture	Millions of IMC patches	Partial	Spatial proteomics not routine clinically	Preclinical
PRISM (2025)	Metabolomics (MS/MS)	CRC biomarker identification	>1B spectra	Growing	CRC metabolomics datasets remain small	Research
MGM / MGM 2.0 (2025)	Microbiome (metagenome)	CRC-associated dysbiosis, FMT response simulation	260 K+ microbiome datasets	Strong	Weak tumor-microbiome pairing in CRC	Preclinical
TransSurv (2023)	Multimodal (WSI + RNA + CNA + clinical)	CRC survival prediction	TCGA-CRC	Partial	No prospective evaluation	Translational preclinical
Subtype-Former (2022)	Multi-omics	CRC subtype discovery	TCGA (multi-omics)	No	Sensitive to missing modalities	Research
xTrimoGene (2023)	Pharmacogenomics	CRC drug-response prediction	Multi-omics drug screen datasets	No	In-vitro bias in drug screens	Research
CancerFoundation (2024)	Single-cell + drug response	Predicts chemotherapy response in CRC	Single-cell perturbation atlases	Partial	Lacks wet-lab validation loop	Early translational

## Synthesizing the data: multimodal foundation models in colorectal cancer

The growing body of work in CRC FMs reveals a clear trajectory, progressing from unimodal predictors that excel within isolated data silos to emerging multimodal architectures that attempt to capture the disease in its full biological and clinical complexity. CRC is not defined by any single layer of biological or clinical information. Epigenomic alterations and DNA mutations propagate RNA expression, shape protein signaling, remodel the TME, and ultimately leave recognizable imprints in histological slides and radiological scans [8]. The movement toward MFMs is, in essence, an attempt to mirror this biological cascade within machine learning frameworks.

Early efforts demonstrated the potential of combining molecular and morphological features. Models such as TransSurv fused WSI with transcriptomic and copy number data to improve survival prediction, demonstrating that morphology and genomics together can yield more refined risk stratification than either modality alone [99]. Building on this, Gao and colleagues introduced a bridged fusion strategy capable of imputing missing modalities, representing a critical advance given the prevalence of incomplete clinical datasets [100]. Similarly, CATfusion scaled multimodal integration to encompass histology alongside a broad spectrum of omic data including mRNA, miRNA, methylation, mutations, and copy number, successfully grouping CRC patients into clinically meaningful subtypes [16]. Graph-based frameworks such as MOSEGCN offered an additional perspective by embedding molecular interactions and patient similarity networks, thereby producing predictions that are both robust and biologically interpretable [29]. Collectively, these examples represent early steps toward models that do not merely summarize data but instead attempt to capture the complex web of relationships underlying CRC heterogeneity.

In parallel, pathology and radiology FMs have matured to a point where they can serve as foundational pillars for CRC multimodal integration. Systems such as UNI [8], PLUTO [101], and TITAN [102] have been trained on millions of histopathology images, learning generic features that transfer across diagnostic tasks, while CT-CLIP [103] has pioneered cross-modal alignment between radiological images and textual data. More recent pathology FMs such as MUSK [104], Virchow [105], and PRISM have further expanded capabilities, supporting biomarker prediction and pan-cancer detection at scale. Although not CRC-specific, these imaging models provide the morphological backbone upon which multimodal integration can be constructed.

At the molecular level, omics-specific FMs are likewise converging toward integrative frameworks. Geneformer, scGPT, RNA-FM [106], and tGPT [107] capture cellular states at single-cell or bulk resolution, while molecular language models such as MolFM [108], GenePT [109], and DNABERT-2 [110] demonstrate that DNA, RNA, and protein sequences can be represented in shared vector spaces. Importantly, integrative multimodal approaches are already emerging in adjacent biomedical domains. Models such as BioMedGPT [111], and BiomedCLIP [112] show that image, text, and molecular data can co-exist within one representational framework, while graph-based methods illustrate how molecular knowledge networks can inform multimodal learning. Though developed at pan-cancer or pan-biology scales, these approaches establish architectural templates for the development of CRC-specific MFMs. Recently developed multimodal and clinical-integration models are summarized in Supplementary Table S7.

The evolution from unimodal models to MFMs illustrates how CRC can be represented more faithfully through the alignment of molecular, morphological, and clinical data streams. Yet, the ultimate significance of these models lies not in technical elegance but in their potential to reshape patient care. The next step is to consider how these integrative architectures move from research into practice and how they can inform diagnosis, prognosis, treatment selection, and real-time monitoring in the clinic. Section 5 explores this clinical frontier, where the promise of CRC MFMs is tested against the practical realities of translation, validation, and patient impact.

## The clinical frontier: translating multimodal foundation models into patient care

If MFMs embody the technical convergence of genomics, pathology, radiology, and clinical data, their ultimate value is measured by their potential to improve patient care. In the context of CRC, this potential is particularly compelling. Clinical decision-making remains fragmented: molecular tests identify subgroups such as MSI-H or RAS mutations, histology informs staging and grading, radiology guides treatment monitoring, and clinical records provide essential contextual information. Each modality provides valuable insight, yet none alone captures the full heterogeneity of CRC. FMs have the potential to unify these perspectives into a single predictive and adaptive framework, thereby reshaping the processes of diagnosis, patient stratification, and treatment selection.

### AI-driven biomarker discovery: from hypothesis generation to validation

FMs act as hypothesis-generating engines, capturing complex molecular and phenotypic patterns that may be overlooked by traditional analyses. By interrogating internal model representations, for instance, using attention maps to identify which genes, image regions, or pathways drive predictions, researchers can uncover novel biomarker candidates. A notable example is the Gene Swin Transformer, which applied this approach to nominate *PEX10* as a previously unrecognized prognostic gene in CRC, subsequently confirmed through experimental validation [61]. Such frameworks go beyond correlative analyses, enabling AI-guided discovery that is mechanistically grounded and clinically relevant.

### Redefining prognostication and molecular subtyping

By integrating histology, multi-omics, and clinical data, MFMs can establish more robust and clinically relevant patient stratification systems than conventional methods. Models such as TransSurv consistently outperform conventional Cox regression models based solely on clinical variables, demonstrating a superior capacity to capture the multi-layered determinants of patient outcome. Likewise, transformer-based clustering frameworks such as Subtype-Former can identify novel molecular subtypes from multi-omics data, potentially outperforming the established CMS classification in predicting prognosis and therapeutic response [113]. Collectively, these advances are reshaping how oncologists conceptualize disease heterogeneity, with direct implications for personalized treatment selection.

### Predicting treatment response

Forecasting therapeutic response is a central goal of clinical FMs. In immunotherapy, reliance on single biomarkers, such as MSI status, has proven inadequate to capture the full complexity of tumor-immune interactions. FMs enable the development of composite predictive signatures. Pathology-based systems such as Virchow can infer MSI-H status directly from H&E slides [105], whereas multimodal models like LORIS integrate genomic and clinical features to generate more accurate immune checkpoint inhibitor (ICI) response [114]. Vision-language models such as MUSK further extend this paradigm by integrating pathology images with information extracted from clinical reports, thereby improving predictive accuracy and interpretability [104].

Chemotherapy and targeted therapy pose equally formidable challenges due to the inherent heterogeneity of CRC. scFMs are uniquely positioned to address this complexity by capturing drug responses at the level of individual tumor cells. For example, CancerFoundation can analyze cell-level heterogeneity in drug sensitivity [115]. Applied to large-scale perturbation datasets such as Tahoe-100M, which profiles multiple CRC cell lines treated with hundreds of drugs, these models reveal transcriptomic signatures of sensitivity and resistance. These insights pave the way for highly personalized chemotherapy regimens [116]. Collectively, these advances represent a shift from reliance on static biomarker toward dynamic, model-driven prediction of treatment efficacy across modalities.

### Moving in functional space: omics-assisted drug discovery and pharmacological modeling

**Figure 2:**
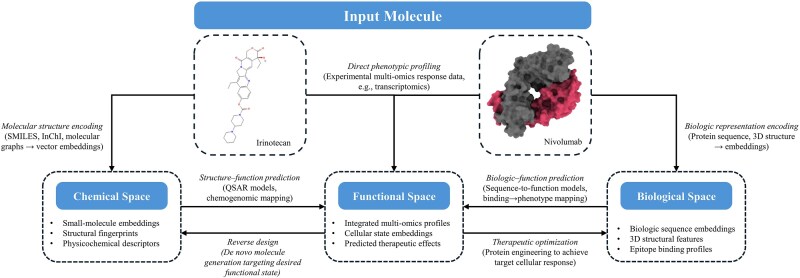
Conceptual framework for therapeutic design using multimodal foundation models (MFMs). Conceptual diagram showing a biological state space where cancer and healthy cell states are mapped, with therapeutic interventions represented as vectors guiding cells from diseased toward healthy states.

Predicting prognoses and treatment responses marks a pivotal step forward, but the ultimate promise of MFMs is not just to interpret the state of a tumor, but to change it. This ambition moves the field from prediction toward therapeutic design, reframing pharmacology as a challenge of navigating a high-dimensional ‘functional space’ [117]. Within this paradigm, MFMs develop computational representation of cellular biology where diseased and healthy states are represented as distinct positions on a complex map. Therapeutic interventions, in this framework, become vector-like transformations capable of steering a cell’s trajectory away from a cancerous state toward a homeostatic one. This shift from descriptive correlation to mechanistic simulation exemplifies the true power of these models (Fig. 2).

This process begins with finding the right therapeutic target. Rather than relying on simple correlations, FMs integrate multi-omic data to uncover the causal drivers of disease. Early approaches such as CellOracle demonstrated how gene regulatory networks reconstructed from single-cell data can be perturbed *in silico* to reveal transcription factors critical for fate decisions [118]. Current FMs extend this concept by integrating proteogenomic and signaling data at scale, enabling the identification of central nodes within CRC networks that mediate resistance, such as compensatory pathways downstream of oncogenic KRAS or aberrant WNT/β-catenin activation [119]. Once a causal target is identified, protein FMs, such as AlphaFold and ESM provide the next crucial step: rapid prediction of a protein’s 3D structure to assess its druggability [120, 121]. For CRC, this is especially important for historically ‘undruggable’ proteins such as β-catenin, where AI-predicted cryptic pockets may reveal novel therapeutic entry points. A model like EMOGI, when applied to CRC data from the CPTAC consortium, could pinpoint kinases like PAK4 as central to compensatory signaling pathways that emerge under targeted therapy, revealing them as high-confidence combination targets [122]. This capacity to infer network-level causality exemplifies the next generation of therapeutic target discovery.

With a viable target identified, the framework transitions to virtual pharmacology, enabling simulation of drug effects prior to laboratory validation. By training on massive perturbation atlases such as Tahoe-100M, scFMs learn to predict the post-treatment transcriptomic state of a cell. Models such as CancerFoundation extend this approach by simulating cell-level heterogeneity in CRC, predicting not only which subpopulations are likely to respond but also those prone to evolve into resistant clones. For instance, in microsatellite-stable (MSS) CRC organoid models, scFMs trained on perturbation data can anticipate the survival of resistant epithelial subclones under MAPK inhibition, demonstrating the ability of virtual pharmacology to flag relapse trajectories before they manifest clinically. In KRAS-mutant MSS tumors, these simulations can predict persistence of resistant states driven by MAPK pathway feedback, thereby guiding rational design of first-line combination regimens that preempt relapse.

Finally, these computational frameworks accelerate the identification of novel drug synergies. Platforms such as xTrimoGene and MTLSynergy analyze large pharmacogenomic screens to predict potential drug interactions [123, 124]. By embedding small-molecule characteristics alongside multi-omic tumor data, these platforms can prioritize combinations most likely to achieve durable therapeutic responses. In CRC, this is particularly critical for treatment-refractory MSS populations, where synergistic strategies such as combining MAPK inhibitors with microenvironment-modulating agents are urgently needed. Pharmacogenomics and drug synergy prediction models relevant to CRC are summarized in Supplementary Table S8.

By integrating causal target discovery, structural assessment, and virtual pharmacology into a single, cohesive framework, MFMs provide a strategy for designing therapies that are mechanistically grounded, patient-specific, and prospectively testable [125]. This integrative approach has the potential to reduce the high attrition rate in drug development and deliver rational, adaptive treatment strategies directly informed by the molecular logic of CRC.

### Closing the loop: experimentally-guided artificial intelligence in colorectal cancer therapeutics

**Figure 3:**
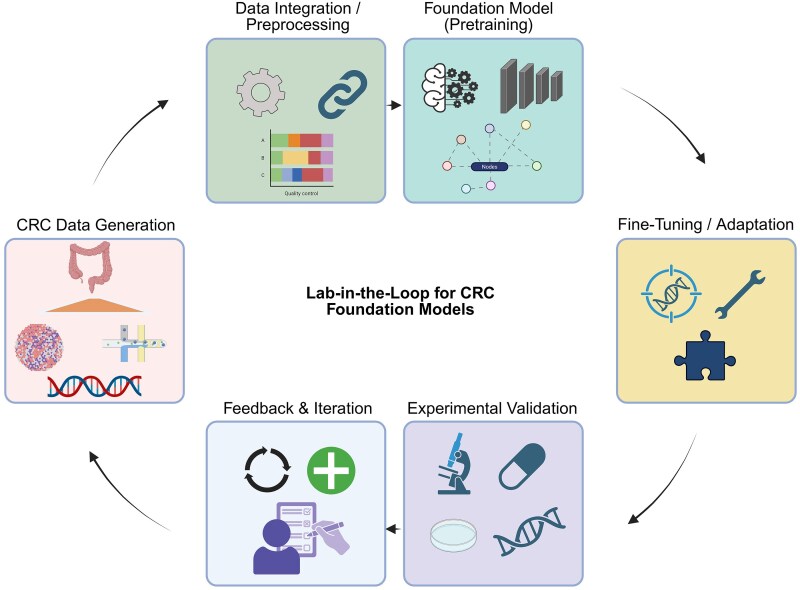
The lab-in-the-loop paradigm for AI-driven biomedical discovery. Diagram showing a feedback loop between AI models and laboratory experiments, where AI-generated hypotheses are tested experimentally and the results are used to improve the models.

While FMs possess the capability to delineate a high-dimensional functional space and replicate pharmacological interventions, their predictive outcomes cannot solely rely on computational methodologies. The ‘lab-in-the-loop’ paradigm is emerging as an essential conduit between *in silico* design and practical application in the real world. In this approach, hypotheses generated by AI such as novel targets, druggable pockets, or synergistic combinations are continuously tested in experimental systems, with the resulting data fed back to refine and retrain the models. This iterative cycle transforms drug discovery from a linear pipeline into a dynamic feedback system (Fig. 3).

Significant initiatives within this domain exemplify its practicality. Genentech has innovated lab-in-the-loop discovery paradigms wherein robotic automation and artificial intelligence collaboratively engage in the design, testing, and iterative refinement of pharmaceutical candidates within a closed-loop system [126]. In a similar vein, NVIDIA has demonstrated AI-enhanced drug discovery platforms that are seamlessly integrated with laboratory automation, highlighting the capacity for foundational models to adapt in near real-time to experimental results [127]. By integrating multi-faceted models with organoid cultures, patient-derived xenografts (PDXs), and high-throughput perturbation assays, CRC research stands to gain distinct advantages from such adaptive cycles. Tumors exhibiting heterogeneous resistance mechanisms, such as KRAS-mutant MSS CRC, serve as optimal test cases where iterative AI-guided design could swiftly lead to the development of rational combination therapies.

For CRC, embedding a lab-in-the-loop framework means that each computational prediction is not an endpoint, but rather the beginning of a refinement process. By aligning virtual pharmacology with automated experimentation, these systems not only accelerate discovery but also enhance trustworthiness by ensuring that AI-driven hypotheses are immediately confronted with biological reality. In doing so, lab-in-the-loop discovery may represent the most tangible pathway through which MFMs can directly shape therapeutic innovation in oncology.

## The path to trustworthy artificial intelligence: challenges and a call for critical evaluation

Despite their immense promise, the path to clinical translation is paved with significant challenges spanning data, modeling, and implementation. Addressing these hurdles is essential for realizing the potential of FMs in routine cancer care. The unresolved domains discussed here are not merely technical limitations but represent fundamental bottlenecks that currently prevent MFMs from closing the loop between computational prediction and actionable clinical intervention.

### Data-related challenges, generalizability, and scale contention

The performance of any FM is fundamentally limited by the quality and scale of its training data, especially when aiming for generalized clinical utility.

- Data scarcity and harmonization: while TCGA is a valuable resource, it lacks sufficient data on rarer but clinically important CRC subtypes. Data generated across different institutions frequently suffer from technical variation (e.g. inconsistent H&E staining protocols) leading to impaired model generalization. This is particularly critical for subtle features like assessing tumor budding at the invasive front of CRC, a key prognostic marker [128].
- Generalizability and calibration: the ‘Foundation’ concept implies broad utility, but robust performance across lower-resource global settings remains largely untested. Despite encouraging performance metrics reported in most studies, there remains a notable paucity of negative, null, or non-replicable findings in the literature. This publication bias limits fair benchmarking, inflates perceived generalizability, and obscures scenarios in which model performance degrades (e.g. domain shift, rare subgroups, low-resource cohorts). Transparent reporting of failures, misclassifications, and poor transferability is therefore essential for a realistic assessment of clinical readiness.

For example, while some DL models predict MSI from WSIs with strong in-domain performance, multi-center external validation has revealed substantial gaps. In a self-supervised, attention-based multi-instance learning study, clinical-grade performance was achieved for MSI and BRAF, while KRAS and NRAS remained ‘clinically insufficient’ in external cohorts [129]. Another hybrid model combining pathology and clinical features achieved an AUC of 0.928 on its internal cohort (TCGA) but dropped to 0.811 in an external cohort [130]. Moreover, systematic reviews have consistently flagged the lack of external validation as a major limitation: many AI-based MSI/KRAS/BRAF prediction studies do not test on independent datasets [131]. Even beyond mutation prediction, a recent DL staging model showed AUC decline from 0.889 internally to 0.700 on external TCGA samples, underscoring how domain-shift and cohort heterogeneity challenge real-world deployment [132]. As FMs move toward clinical integration, rigorous validation across diverse populations, institutions, sequencing or imaging platforms, and disease stages will be critical to accurately determine robustness and true translational potential.

- Contention on scale: it remains unresolved whether billion-parameter, generic MFMs are required for CRC, or whether smaller, specialized multimodal expert models (MEMs) could achieve superior performance on specific clinical endpoints (e.g. MSI prediction) at a fraction of the cost.
- Privacy and governance: the use of sensitive patient data requires robust governance. Techniques like federated learning offer a promising solution to train a shared CRC prognostication model across hospitals in different countries without sharing raw patient data [133].

### Model-related challenges: trust, transparency, and algorithmic bias

The complexity of FMs introduces difficulties related to interpretation, algorithmic integrity, and capturing the dynamic nature of disease.

- The challenge of causal and dynamic inference: current MFMs primarily function as sophisticated predictive models, often excelling at correlation while remaining limited in causal inference (e.g. determining ‘why’ a tumor feature drives resistance). Most MFMs are trained on static patient snapshots, limiting their ability to model the dynamic, evolving nature of CRC such as metastasis initiation, immune evasion, and clonal evolution under therapy.
- Interpretable rationale versus explainable feature: The ‘black box’ nature is a major barrier. Current eXplainable AI (XAI) methods often highlight ‘where’ the model looked (patch-level features) but rarely provide an actionable biological rationale that aligns with physician intuition and regulatory expectations (e.g. linking prediction to known pathway biology) [134, 135]. This partial interpretability raises the risk of automation bias and clinician over-reliance.
- Robustness and bias: rigorous external validation on independent, multi-institutional, and prospective cohorts is the gold standard [136]. If training data is not representative, models can exacerbate health disparities. For example, an AI model for CRC detection trained primarily on light-skinned patient colonoscopy images may exhibit reduced sensitivity for detecting subtle flat polyps in patients with darker skin tones [137].
- Epigenomic integration: the current MFM landscape is image- and sequence-dominant. The vital role of epigenomics (e.g. DNA methylation) in CRC plasticity and CIMP classification is underrepresented. Integrating sparse, high-dimensional regulatory data with dense histology or radiology signals remains a major technical challenge.

### Clinical and regulatory translation

Bridging the gap from a research model to a clinical tool involves overcoming formidable implementation and approval hurdles.

- Regulatory pathways: AI tools are medical devices requiring regulatory approval. The path for ‘continually learning’ models that adapt over time, which would be ideal for dynamic CRC risk models, is still being defined and presents a major regulatory challenge.
- Workflow integration: a model must fit seamlessly into the clinic. A successful CRC pathology AI tool must integrate with the laboratory information system (LIS), present results within the pathologist’s existing digital viewer, and allow them to seamlessly accept, reject, or modify the AI’s findings in their final report.
- Cost-effectiveness and reimbursement: for adoption, these tools must demonstrate value. An AI that predicts MSI status from a routine H&E slide could be highly cost-effective by reducing the number of patients who need expensive follow-up IHC and PCR testing, but this will require formal health economic evaluations to secure reimbursement from payers.

### Future directions and the research agenda

The field is moving rapidly, with several key trends shaping the next generation of models that aim to resolve the current challenges.

- Architectural innovation: the rise of highly efficient architectures like state space models may enable the processing of entire genomes as a single input, potentially capturing
- complex structural variants and long-range epigenetic effects that are missed by current models in CRC [33, 138].
- The ‘Foundationless’ debate and critical evaluation: this debate highlights the need to rethink pre-training strategies and benchmarks for genomics, moving toward more biologically meaningful objectives, as some smaller, well-designed models can outperform massive pre-trained models for certain tasks [139].
- Continual learning for evolving data: suture models will need to incorporate continual learning strategies, such as instruction-based knowledge distillation, to evolve over time without constant retraining from scratch [140], overcoming the ‘catastrophic forgetting’ of previous knowledge when updated with new clinical data.
- Causal inference and digital twins: moving beyond correlation to causation is a critical next step. Causal DL, using frameworks like do-calculus integrated with genomic data, aims to differentiate genes that are merely associated with CRC prognosis from those that mechanistically drive it [141]. This paves the way for the ultimate vision: a ‘digital twin’ of a patient’s tumor, used to simulate its response to different chemotherapy regimens [142].

## Conclusion

MFMs represent more than an incremental advance in CRC; they signal a paradigm shift. By capturing the multilayered interplay of epigenomic and genomic alterations, transcriptomic programs, TME dynamics, and imaging features, MFMs hold the potential to unify previously fragmented approaches to diagnosis and therapy. CRC, with its well-defined yet heterogeneous subtypes such as MSI-H and MSS tumors, provides a uniquely challenging proving ground where the generalizability of oncology FMs can be rigorously tested.

The promise of these models extends beyond prognostic stratification. By embedding disease states within high-dimensional functional spaces, MFMs enable a new computational pharmacology where therapeutic interventions are modeled as transformations of cellular trajectories. This reorientation from static biomarkers to dynamic, mechanistic simulations offers the possibility of rational, adaptive treatment design. The lab-in-the-loop paradigm further accelerates translation by iteratively coupling computational hypotheses with experimental validation, ensuring that AI-driven insights remain grounded in biological reality.

For such advances to impact patient care, trustworthiness must be treated as a cornerstone rather than an afterthought. Fairness, transparency, robustness, and accountability are essential for building confidence among clinicians and patients alike. Only models that can withstand rigorous external validation, explain their reasoning in clinically interpretable terms, and avoid exacerbating health inequities will be acceptable for integration into oncology practice.

Realizing this vision requires deep interdisciplinary collaboration. Computer scientists, systems biologists, experimental researchers, and clinicians must converge on shared frameworks that support both technical innovation and clinical feasibility. By embedding trustworthy AI within a lab-in-the-loop translational ecosystem, CRC research can set the precedent for how MFMs are deployed across oncology. The challenge now is not only to refine the models but to build the collaborative infrastructure that will carry them from computational elegance to clinical impact.

**Key Points**

- Colorectal cancer (CRC) is marked by profound molecular and clinical heterogeneity that limits the utility of single-modality biomarkers; multimodal foundation models (MFMs) provide a unified framework to capture this complexity.
- By integrating histopathology, radiology, multi-omics, and clinical records, MFMs enable accurate prognostication, molecular subtyping, and treatment response prediction that surpass conventional approaches.
- Beyond prediction, MFMs can simulate disease dynamics and therapeutic perturbations within high-dimensional ‘functional spaces’, supporting rational drug discovery and adaptive treatment design.
- The lab-in-the-loop paradigm, coupling artificial intelligence predictions with experimental validation, is central to building trustworthy, clinically actionable MFMs in CRC.
- Trustworthy translation requires interpretability, equity, and rigorous multi-institutional validation to ensure safe integration of MFMs into oncology practice.

## Supplementary Material

Supplementary_Tables_bbag179

## Data Availability

No new data were generated or analyzed in this study. All information presented is derived from previously published studies cited within the manuscript.

## Organization description

### Cellular and molecular research center

The Cellular and Molecular Research Center at Birjand University of Medical Sciences conducts interdisciplinary research in molecular biology, cancer research, and translational medicine, with a focus on integrating experimental and computational approaches.
