# Supplementary material for: Multimodal foundation models in colorectal cancer: from prediction to trustworthy clinical insight
Source: Brief Bioinform. 2026 Apr 27;27(2):bbag179. doi: 10.1093/bib/bbag179 (PMC13116344; doi:10.1093/bib/bbag179)
Supplement: Supplementary_Tables_bbag179 [file supplementary_tables_bbag179.docx]

**Supplementary Tables:**

**Table S1: Histopathology & Radiology:** FMs for whole-slide histopathology and radiology in CRC, including architectures, training scale, typical applications, and source links.

| Model | Year | Params (approx) | Modality / Scope | Typical CRC Applications | GitHub | Notes / Sources |
| --- | --- | --- | --- | --- | --- | --- |
| UNI | 2024 | Not specified (ViT encoder) | Histopathology FM, pre-trained on ~100K WSIs / ~100M patches | WSI feature extraction, MSI prediction, outcome modeling, cross-hospital transfer | [mahmoodlab/UNI](https://github.com/mahmoodlab/UNI) | Nature Medicine paper + repo & HF model cards. (GitHub, Hugging Face) |
| CONCH | 2024 | Not specified (CLIP-style encoders) | Vision–Language FM for pathology (1.17M image-caption pairs) | Zero-shot captioning & retrieval on H&E; improves biomarker prediction when paired with text | [mahmoodlab/CONCH](https://github.com/mahmoodlab/CONCH) | Paper & model card describe dataset scale and VLM design. (GitHub, PMC, Hugging Face) |
| TITAN | 2024 | Not specified | Multimodal WSI FM (self-supervision + vision-language alignment; 335,645 WSIs) | WSI representation learning; report-aware retrieval; robust transfer | [mahmoodlab/TITAN](https://github.com/mahmoodlab/TITAN) | Pretrained with paired reports & synthetic captions. GitHub |
| RetCCL | 2023 | ResNet-based (not stated) | Self-supervised WSI features (contrastive, clustering-guided) | Patch features for MIL (CRC grading, biomarker classification), WSI retrieval | [Xiyue-Wang/RetCCL](https://github.com/Xiyue-Wang/RetCCL) | MIA paper + results incl. a colorectal dataset benchmark. GitHub |
| HIPT | 2022 | ViT-H/ViT-B (not stated) | Hierarchical image pyramid transformer for WSIs | Multi-scale WSI encoding for CRC subtyping & prognosis (downstream MIL) | [mahmoodlab/HIPT](https://github.com/mahmoodlab/HIPT) | Original repo/paper for hierarchical transformer. |
| CLAM | 2020 | MIL framework (backbone-dependent) | Weakly supervised MIL for WSIs | Slide-level CRC tasks (tumor/normal, MSI) using patch features | [mahmoodlab/CLAM](https://github.com/mahmoodlab/CLAM) | Classic MIL baseline widely adopted. |
| BiomedCLIP | 2023 | ViT-B/16 (86M) + PubMedBERT (110M) | Biomedical vision–language (15M figure-caption pairs) | Radiology report grounding, cross-modal retrieval (CRC imaging/figures) | [microsoft/BiomedCLIP](https://huggingface.co/microsoft/BiomedCLIP-PubMedBERT_256-vit_base_patch16_224) | Paper + HF model card. (arXiv, Hugging Face) |
| RadImageNet | 2022 | CNN backbones (e.g., ResNet, DenseNet) | Radiology pretraining (CT/MRI/US, 1.35M images) | Transfer learning for abdominal/pelvic CT, liver lesions, CRC staging tasks | [BMEII-AI/RadImageNet](https://github.com/BMEII-AI/RadImageNet) | Dataset & transfer results. (PMC, GitHub) |
| MedSAM | 2023-24 | SAM ViT-H (~632M) adapted | “Segment Anything” adapted to medical images | Organ/tumor segmentation in CT/MRI/WSI figures (pre/post-op CRC planning) | [bowang-lab/MedSAM](https://github.com/bowang-lab/MedSAM) | Nature Comms paper & official repo. (Nature, GitHub) |
| MUSK | 2025 | Not specified (Multimodal Transformer) | Vision-language FM for precision oncology (unpaired pathology images and text) | Biomarker prediction (e.g., MSI, mutations) and survival analysis in CRC and other cancers | [lilab-stanford/MUSK](https://github.com/lilab-stanford/MUSK) | Integrates vision-language pretraining with unified mask modeling. |
| KEEP | 2025 | Not specified | Knowledge-enhanced pathology vision-language FM | Cancer diagnosis, including CRC subtype classification and biomarker discovery | [MAGIC-AI4Med/KEEP](https://github.com/MAGIC-AI4Med/KEEP) | Incorporates disease knowledge graphs into VLM pretraining for improved interpretability. |
| Prov-GigaPath | 2024 | Not specified | Whole-slide FM from real-world data (~1.3B image tiles) | Prognostic modeling and biomarker prediction in CRC WSIs | [prov-gigapath/prov-gigapath](https://github.com/prov-gigapath/prov-gigapath) | Pretrained on gigapixel WSIs; strong transfer to CRC tasks. Nature paper. |
| Virchow | 2024 | Not specified (Vision Transformer) | Self-supervised vision transformer (pretrained on 1.5M WSIs) | Pan-cancer detection, including CRC grading and MSI prediction | [paige-ai/Virchow](https://huggingface.co/paige-ai/Virchow) | Foundation model for clinical-grade pathology; benchmarked on CRC cohorts. |
| H-optimus-0 | 2024 | Not specified | Histopathology FM (self-supervised) | Tissue classification and outcome prediction in CRC | [bioptimus/H-optimus-0](https://huggingface.co/bioptimus/H-optimus-0) | Open-source foundation model for histology; transferable to CRC datasets. |
| DeepCMorph | 2024 | Not specified | Cell morphology-aware CNN for histopathological images | CRC tissue classification (e.g., NCT-CRC-HE dataset with 96.99% accuracy) | [aiff22/DeepCMorph](https://github.com/aiff22/DeepCMorph) | Dual-module (nuclei segmentation + tissue classification); pretrained on pan-cancer TCGA. |
| CPath (New) | 2024 | ViT-L + ClinicalBERT | Vision-Language FM for pathology trained on text-grounded images | Zero-shot classification of CRC subtypes, visual question answering on CRC histology | [kyunghun/CPath](https://www.google.com/search?q=https://github.com/kyunghun/CPath) | A large-scale VLM trained on 1.6B image patches from textbooks and reports. |
| Quilt-1M | 2024 | ViT-L + PubMedBERT | Vision-Language model and dataset (1M image-text pairs) | Cross-modal retrieval and classification for CRC and other pathology images | [geva-on-github/Quilt-1M](https://www.google.com/search?q=https://github.com/geva-on-github/Quilt-1M) | Large-scale public dataset and VLM to advance open science in pathology. |
| Wagner Pipeline | 2023 | Not specified (Transformer) | Histopathology (WSI) | Predicts MSI, RAS/BRAF mutations, CMS from CRC slides | (Not public) | Multicenter CRC cohort, 13k patients. |
| ROSIE | 2025 | ConvNext backbone | Histopathology FM (~16M cells, H&E+mIF) | In silico mIF prediction, TME analysis in CRC | [enable-medicine-public/rosie](https://gitlab.com/enable-medicine-public/rosie) | Ochsner-CRC dataset validation. |
| CHIEF | 2024 | Not specified | Histopathology FM | Infers molecular features (e.g., BRAF mutations) from H&E in CRC | [hms-dbmi/CHIEF](https://github.com/hms-dbmi/CHIEF) | H&E to genomics inference. |
| PLUTO | 2024 | Not specified | WSI pathology FM | Pan-cancer pathology FM, transfer to CRC | (Not public) | CRC biomarker prediction baseline. |

**Table S2: Genomics (DNA/Regulatory):** DNA-based FMs for regulatory genomics and variant effect prediction, with emphasis on CRC-relevant applications.

| Model | Year | Params (approx) | Application Focus | Typical CRC Uses | GitHub | Notes / Sources |
| --- | --- | --- | --- | --- | --- | --- |
| DNABERT | 2021 | BERT-base (~110M) | k-mer BERT for DNA sequences | Motif discovery in CRC drivers; enhancer/promoter classification | [jerryji1993/DNABERT](https://github.com/jerryji1993/DNABERT) | Original repo. |
| DNABERT-2 | 2023 | 110M | Efficient multi-species genome foundation model | Improved performance on promoter and splice site prediction relevant to CRC driver genes | [MAGICAL-Lab/DNABERT-2](https://www.google.com/search?q=https://github.com/MAGICAL-Lab/DNABERT-2) | arXiv preprint; more efficient pre-training and broader species coverage. |
| Enformer | 2021 | ~216M | Long-range sequence → gene expression/epigenomic profiles | Non-coding variant effect prediction for CRC risk loci | [deepmind/enformer](https://github.com/deepmind/deepmind-research/tree/master/enformer) | Official implementation & paper. |
| Nucleotide Transformer | 2023 | up to billions (various sizes) | Large-scale masked LMs for DNA across species | Transfer to regulatory element prediction in colon tissue | [instadeepai/nucleotide-transformer](https://github.com/instadeepai/nucleotide-transformer) | Paper & repo. |
| HyenaDNA | 2023 | varies (not stated) | Long-range genomic modeling (Hyena operators) | Enhancer–promoter linking, TAD-scale features in CRC | [HazyResearch/hyena-dna](https://github.com/HazyResearch/hyena-dna) | Long-context DNA modeling. |
| GENA-LM | 2024 | multi-size checkpoints | Foundation LMs for genomes/proteins/chem | Broad genomic representation learning; variant effect transfer | [microsoft/GENA-LM](https://www.google.com/search?q=https://github.com/microsoft/GENA-LM) | MSR umbrella repo. |
| Evo | 2024 | Not specified (StripedHyena architecture) | Biological foundation modeling from molecular to genome scale | Variant effect prediction and regulatory modeling in CRC genomes | [evo-design/evo](https://www.google.com/search?q=httpshttps://github.com/evo-design/evo) | Long-context genomic FM; enables sequence design across domains. BioRxiv paper. |
| dnaGrinder | 2024 | 140M | Lightweight, high-capacity genomic foundation model | Variant effect prediction, species identification, and regulatory element annotation | [Weng-lab/dnaGrinder](https://www.google.com/search?q=https://github.com/Weng-lab/dnaGrinder) | arXiv preprint; designed for efficiency and broad applicability. |
| AlphaMissense | 2023 | Not specified (Transformer-based) | Missense variant pathogenicity prediction | Prioritizing functional missense mutations (e.g., in *APC*, *KRAS*, *TP53*) from CRC sequencing data | [google-deepmind/alphamissense](https://github.com/google-deepmind/alphamissense) | State-of-the-art for classifying variants; scores for all human missense variants available. Science paper. |
| Caduceus | 2024 | 125M - 1.3B | Mamba-based long-context (1M tokens) DNA model | Capturing long-range regulatory interactions (e.g., distal enhancers) that drive CRC oncogenes | [google-deepmind/caduceus](https://www.google.com/search?q=https://github.com/google-deepmind/caduceus) | BioRxiv preprint; new architecture for genome-scale modeling. |
| AlphaGenome | 2025 | Not specified | Genomics FM | Variant effect prediction of non-coding CRC mutations | [google-deepmind/alphagenome](https://github.com/google-deepmind/alphagenome) | DeepMind, 2025. |

**Table S3: Transcriptomics (Bulk & Single-Cell/Spatial):** Transcriptome FMs spanning bulk, single-cell, and spatial modalities, applied to CRC tumor biology and the TME.

| Model | Year | Params (approx) | Application Focus | Typical CRC Uses | GitHub | Notes / Sources |
| --- | --- | --- | --- | --- | --- | --- |
| scGPT | 2023-25 | Not specified (Transformer) | Foundation model for single-cell multi-omics; 33M+ cells; spatial continual pretraining (scGPT-spatial) | Cell type/state inference in CRC TME; spatial mapping from Visium/Slide-seq | [bowang-lab/scGPT](https://github.com/bowang-lab/scGPT) | Repo; docs; spatial extension. (GitHub, scgpt.readthedocs.io) |
| Geneformer | 2023-25 | ~120M (encoder) | Transformer pre-trained on ~30M single cells (CELLxGENE census) | Zero-shot/transfer features for CRC scRNA; perturbation inference | [jkobject/geneformer](https://github.com/jkobject/geneformer) | Repo + NVIDIA page + updates. (GitHub, NVIDIA GitHub, Hugging Face) |
| scFoundation | 2024-25 | Not specified | Large-scale single-cell FM (BioMap/Tsinghua/MBZUAI) | General embeddings for CRC atlas studies; batch integration | [biomap-research/scFoundation](https://github.com/biomap-research/scFoundation) | Repo & HF card. (GitHub, Hugging Face) |
| scBERT | 2021-22 | BERT-base-like | Pretrained LM for cell-type annotation from scRNA-seq | Automated CRC cell-type labeling; tumor vs stromal immune subsets | [TencentAILabHealthcare/scBERT](https://github.com/TencentAILabHealthcare/scBERT) | Paper & follow-up reusability note. (GitHub, BioRxiv) |
| CellFM | 2025 | ~800M | Large FM trained on 100M cells (RetNet-based) | High-capacity embeddings for complex CRC tissues | [biomed-AI/CellFM](https://github.com/biomed-AI/CellFM) | Nature Communications 2025. Nature |
| GIST | 2024 | Not specified | Spatially resolved transcriptomics (gene imputation and spatial transcriptomics) | Gene expression pattern measurement in CRC tissues at subcellular resolution | [lengjk1214/GIST](https://github.com/lengjk1214/GIST) | Focuses on spatial transcriptomics; improves resolution in CRC samples. |
| TranscriptFormer | 2025 | Not specified (Generative Transformer) | Cross-species single-cell FM (>112M cells, 12 species) | Zero-shot annotation of CRC cell types; contextualizing human TME within broader evolutionary frameworks | [karaletsos-lab/transcriptformer](https://www.google.com/search?q=https://github.com/karaletsos-lab/transcriptformer) | BioRxiv preprint; massive scale enables robust transfer learning. |
| scCello | 2024 | Not specified (Transformer) | Cell ontology-guided transcriptome FM (22M cells) | Enhanced zero-shot identification of rare CRC immune subtypes; predicting marker genes | [snap-stanford/scCello](https://www.google.com/search?q=https://github.com/snap-stanford/scCello) | NeurIPS paper; incorporates biological hierarchy for improved generalization. |
| Cell2Sentence | 2024 | Not specified (uses general LLMs) | Framework to represent single-cell data as "sentences" for LLMs | Interrogating CRC cell states and drug responses using natural language queries | [dvanleuven/cell2sentence](https://www.google.com/search?q=https://github.com/dvanleuven/cell2sentence) | BioRxiv preprint; bridges single-cell biology with the power of LLMs. |
| STFormer | 2025 | Not specified (Vision Transformer) | Infers spatial gene expression from H&E images | Creating "virtual" spatial omics maps from routine CRC pathology slides | [caoshirui/STFormer](https://www.google.com/search?q=https://github.com/caoshirui/STFormer) | BioRxiv preprint; a cross-modal model bridging histology and transcriptomics. |
| Nicheformer | 2024 | Not specified (Transformer) | Identifies multicellular functional neighborhoods (niches) from spatial omics | Modeling tumor-immune interactions and immunosuppressive niches in the CRC TME | [theislab/nicheformer](https://github.com/theislab/nicheformer) | BioRxiv preprint; explicitly models cell-cell interactions in their spatial context. |
| Gene Swin Transformer | 2025 | Not specified (Swin Transformer) | Converts transcriptomics into synthetic images | Prognosis prediction, PEX10 biomarker in CRC | - | Novel AI method. |
| scPRINT | 2025 | Not specified | Pretrained on >50M cells | Infers GRNs, dysfunctional signaling in CRC | [cantinilab/scPRINT](https://github.com/cantinilab/scPRINT) | scFM for CRC networks. |

**Table S4: Proteomics / Structure & Design:** Protein language and generative structure models relevant to CRC, including applications in function prediction, drug discovery, and spatial proteomics.

| Model | Year | Params (approx) | Application Focus | Typical CRC Uses | GitHub | Notes / Sources |
| --- | --- | --- | --- | --- | --- | --- |
| ESM-2 | 2022 | 8M-15B (family) | Protein language model (masked LM) | CRC variant effect prediction; protein function | [facebookresearch/esm](https://github.com/facebookresearch/esm) | Meta AI repo. |
| ProtTrans / ProtT5 | 2020-21 | up to ~3B | Protein LMs (BERT/T5 family) | Function/loc prediction for CRC-related proteins | [agemagician/ProtTrans](https://github.com/agemagician/ProtTrans) | Collection of pretrained protein LMs. PMC |
| AlphaFold2 | 2021 | Not applicable | Structure prediction from sequence | Mutational impact on CRC proteins; drug target triage | [deepmind/alphafold](https://github.com/deepmind/alphafold) | Official code release. PMC |
| RFdiffusion | 2023 | Not specified | Protein design by diffusion (RosettaFold-based) | Designing binders to CRC targets (e.g., EGFR variants) | [RosettaCommons/RFdiffusion](https://github.com/RosettaCommons/RFdiffusion) | Official repo. (BioRxiv) |
| VirTues | 2025 | Not specified | Foundation model for multiplexed tissue imaging (spatial proteomics across scales) | Clinical diagnostics and biological discovery in CRC tissues | [bunnelab/virtues](https://github.com/bunnelab/virtues) | Analyzes molecular, cellular, and tissue scales; outperforms baselines in cancer tasks. |
| AlphaFold 3 | 2024 | Not specified (Diffusion-based) | Structure prediction for all life's molecules (proteins, DNA, RNA, ligands) | Modeling KRAS-drug interactions, DNA-binding of transcription factors like β-catenin | [deepmind/alphafold](https://github.com/deepmind/alphafold) | A new generative architecture that dramatically expands predictive scope. Nature paper. |
| ESM3 | 2024 | Not specified | Generative protein language model and structure predictor | Generating novel functional proteins; high-resolution structure prediction of CRC-related enzymes | [facebookresearch/esm](https://github.com/facebookresearch/esm) | Meta AI repo; new model family with improved generative and structural capabilities. |
| ProtGPT2 | 2022 | ~738M | Deep unsupervised language model for protein design | *De novo* design of peptides or small proteins to inhibit CRC signaling pathways | [nferruz/ProtGPT2](https://www.google.com/search?q=https://github.com/nferruz/ProtGPT2) | Nature Comms paper; applies GPT-2 architecture to protein sequence generation. |
| KRONOS | 2025 | Not specified | FM for spatial proteomics | Segmentation-free cell phenotyping and patient stratification from CRC tissue imaging mass cytometry data | [mahmoodlab/KRONOS](https://github.com/mahmoodlab/KRONOS) | arXiv preprint; a powerful tool for analyzing spatial organization in CRC. |
| EMOGI | 2021 | Not specified | Proteogenomics / GNN | Identifies CRC drug targets downstream of KRAS | [EMOGI: An explainable multi-omics graph integration method based on graph convolutional networks to predict cancer genes.](https://github.com/schulter/EMOGI) | Applied to CPTAC CRC data. |

**Table S5: Metabolomics (MS/MS) & Chem:** Metabolomics and cheminformatics FMs for CRC biomarker identification, metabolite annotation, and therapeutic chemical space exploration.

| Model | Year | Params (approx) | Application Focus | Typical CRC Uses | GitHub | Notes / Sources |
| --- | --- | --- | --- | --- | --- | --- |
| MS2Transformer | 2021-22 | Not specified | Transformer for MS/MS spectral annotation | CRC metabolite ID in stool/serum MS | [HuberGroup-EMBL/ms2transformer](https://www.google.com/search?q=https://github.com/HuberGroup-EMBL/ms2transformer) | Repo for transformer-based MS/MS. |
| MS2DeepScore | 2021 | Not specified | Learned similarity for spectra | Candidate ranking for CRC biomarkers | [matchms/ms2deepscore](https://github.com/matchms/ms2deepscore) | Official repo. |
| ChemBERTa | 2020 | RoBERTa variants | SMILES language models | QSAR/tox for CRC drug screening | [seyonechithrananda/chemberta](https://www.google.com/search?q=https://github.com/seyonechithrananda/chemberta) | Original repo. |
| DreaMS | 2025 | Not specified | Self-supervised learning of molecular representations from MS/MS spectra | Metabolite identification and chemical property prediction in CRC samples | [pluskal-lab/DreaMS](https://github.com/pluskal-lab/DreaMS) | Pretrained on 201M unannotated MS/MS spectra; includes DreaMS Atlas. Nature Biotechnology paper. |
| PRISM | 2025 | Not specified (Transformer) | Foundation model for life's chemistry (trained on >1B spectra) | High-throughput annotation of metabolic biomarkers in CRC; identifying novel natural products | [enveda/prism](https://enveda.com/prism-a-foundation-model-for-lifes-chemistry/) | Enveda Biosciences announcement; a large-scale FM for metabolomics. |
| LSM1-MS2 | 2024 | Not specified (Transformer) | Self-supervised FM for tandem MS applications | *De novo* molecular generation and robust metabolite annotation from CRC metabolomic profiles | (Code not yet public) | arXiv preprint; demonstrates strong performance in low-data regimes. |
| MassFormer | 2021 | Not specified (Graph Transformer) | Predicts MS2 spectra from molecular graphs | Improving confidence of metabolite identification in CRC by matching experimental vs. predicted spectra | [h-rost/massformer](https://www.google.com/search?q=https://github.com/h-rost/massformer) | arXiv preprint; learns atom-bond relationships to predict fragmentation. |
| MS2Mol | 2023 | Not specified (Transformer) | *De novo* structural prediction from mass spectra | Elucidating the structure of unknown, potentially novel, metabolic biomarkers found in CRC patients | [butler-lab/MS2Mol](https://www.google.com/search?q=https://github.com/butler-lab/MS2Mol) | arXiv preprint; illuminates "dark chemical space" without relying on reference libraries. |

**Table S6: Microbiomics & Metagenomics:** FMs for microbiome and metagenomic analyses, highlighting their role in CRC-associated microbial communities and functional inference.

| Model | Year | Params (approx) | Application Focus | Typical CRC Uses | GitHub | Notes / Sources |
| --- | --- | --- | --- | --- | --- | --- |
| MICAH | 2024 | Not specified (two-layer Graph Transformer) | Heterogeneous graph transformer that integrates abundance, phylogenetic, and metabolic data | Discovering cancer-associated microbial communities (rather than isolated taxa) relevant to CRC initiation and progression | [OSU-BMBL/micah](https://github.com/OSU-BMBL/micah) | An explainable graph neural framework to identify cancer-associated intratumoral microbial communities |
| IMPACT | 2024 | Not specified (CNN-based) | Applies computer vision techniques (saliency mapping) to microbial abundance data | Highlighting key microbial taxa and their functional attributes that are predictive of CRC risk | [SydneyBioX/IMPACT](https://github.com/SydneyBioX/IMPACT) | An interpretable microbial phenotype analysis framework that treats microbial data as image-like representations |
| MGM (Microbial General Model) | 2025 | Millions of parameters (Transformer) | FM for microbiome analyses that treats samples as "sentences" and taxa as "words" | Disease classification, biomarker discovery, and analysis of microbiome-host interactions relevant to CRC | [HUST-NingKang-Lab/MGM](https://github.com/HUST-NingKang-Lab/MGM) | Pretrained on over 260,000 microbiome samples to capture complex inter-species interactions |
| MGM 2.0 | 2025 | Not specified (Generative Transformer) | Extends MGM for generative microbiome modeling | Simulating therapeutic microbiome engineering for CRC, such as predicting fecal microbiota transplantation (FMT) outcomes | [HUST-NingKang-Lab/MGM](https://github.com/HUST-NingKang-Lab/MGM) | Capable of colonization prediction and disease-conditioned microbiome synthesis |
| gLM (Genomic Language Model) | 2024 | Not specified (RoBERTa-based) | Contextualizes microbial genes by modeling their organization on the contig scale | Analyzing differential gene functions in CRC-associated microbes; identifying mobile genetic elements related to dysbiosis | [y-hwang/gLM](https://github.com/y-hwang/gLM) | Uses ESM-2 embeddings for individual genes and models their relationships within their genomic neighborhood |
| FGBERT | 2024 | Not specified (Transformer) | Function-driven pre-trained language model for metagenomic sequences | Downstream prediction of gene operons, functional pathways, and nitrogen cycles within the CRC metagenome | [idrugLab/FG-BERT](https://github.com/idrugLab/FG-BERT) | The first metagenomic pre-trained model designed to encode both context-aware and function-relevant gene representations |
| ProkBERT family | 2024 | Not specified (Encoder-only) | Genomic language models specifically designed for microbiome applications using Local Context-Aware (LCA) tokenization | Downstream tasks relevant to the CRC microbiome, such as bacterial promoter prediction and bacteriophage identification | [nbrg-ppcu/prokbert](https://github.com/nbrg-ppcu/prokbert) | A family of models focused on generating nucleotide sequence representations for prokaryotic genomes |
| ViraLM | 2024 | Not specified (DNABERT-2 adapted) | A viral language model fine-tuned for the binary classification of novel viral contigs in metagenomic data | Identifying and annotating bacteriophages in the CRC gut virome, which are critical components of gut health | [ChengPENG-wolf/ViraLM](https://github.com/ChengPENG-wolf/ViraLM) | Adapts the genome foundation model DNABERT-2 for virus detection, showing improved performance over training from scratch |
| BGC-Prophet | 2023 | Not specified (Transformer) | A neural network model that uses NLP techniques to predict BGCs | Mining the CRC microbiome for novel secondary metabolites with potential anti-cancer or therapeutic properties | [HUST-NingKang-Lab/BGC-Prophet](https://github.com/HUST-NingKang-Lab/BGC-Prophet) | Uses ESM-2 embeddings and processes amino acid sequences to detect patterns indicative of BGCs |

**Table S7: Multimodal & Clinical Integration Models:** FMs integrating pathology, omics, and clinical features for CRC prognosis, subtype discovery, and therapy stratification.

| Model | Year | Params (approx) | Modality / Scope | Typical CRC Applications | GitHub / Source | Notes / Sources |
| --- | --- | --- | --- | --- | --- | --- |
| TransSurv | 2023 | Not specified | Multimodal fusion (Histopathology WSIs + RNA-seq + Copy-Number Alterations + Clinical features) | Prognosis and survival prediction in CRC through cross-modal transformer fusion | [stc04003/tranSurv](https://github.com/stc04003/tranSurv) | Outperforms baseline models on TCGA-CRC cohorts by capturing intra- and inter-modality interactions. |
| Subtype-Former | 2022 | Not specified | Transformer-based multi-omics representation learning | Unsupervised CRC subtype discovery using TCGA multi-omics data; improved stratification compared to classical clustering | [haiyangLab/Subtype-Former](https://github.com/haiyangLab/Subtype-Former) | Leverages multi-omics Transformer with MLP head for cancer subtype identification. |
| LORIS | 2024 | Not applicable (logistic regression model) | Clinical + genomic (6 feature signature: age, sex, TMB, MSI, histology, PD-L1) | Stratifies CRC and pan-cancer patients for immune checkpoint blockade response and survival | [LORIS: LORIS: A LOgistic Regression-based Immunotherapy-response Score](https://github.com/rootchang/LORIS) | Simple interpretable predictor outperforming TMB and PD-L1 biomarkers. |

**Table S8: Pharmacogenomics & Drug Synergy Models:** FMs for pharmacogenomics and drug synergy prediction, enabling CRC drug response modeling and therapeutic optimization.

| Model | Year | Params (approx) | Modality / Scope | Typical CRC Applications | GitHub / Source | Notes / Sources |
| --- | --- | --- | --- | --- | --- | --- |
| xTrimoGene | 2023 | Not specified (Transformer-based) | Pharmacogenomics FM trained on large-scale gene expression + drug perturbation data | Predicts CRC drug response and drug–gene interactions; prioritizes therapeutic candidates | - | Demonstrates strong transfer to CRC pharmacogenomic datasets and biomarker discovery. |
| MTLSynergy | 2022 | Not specified (Multi-task Transformer) | Predicts drug synergy scores across cancer cell lines by integrating gene expression, mutations, and drug fingerprints | Identifies effective CRC drug combinations and synergistic therapies | [TOJSSE-iData/MTLSynergy](https://github.com/TOJSSE-iData/MTLSynergy) | Multi-task learning framework tested on GDSC/CTRP datasets, including CRC lines. |
| DrugCell | 2020 | ~10M (deep neural network with biological hierarchy) | Interpretable pharmacogenomic model linking gene mutations to drug response | CRC cell line and patient-level drug sensitivity prediction; explains mechanism of resistance | [idekerlab/DrugCell](https://github.com/idekerlab/DrugCell) | Widely cited benchmark model for drug response prediction. |
| CancerFoundation | 2024 | Not specified (Transformer) | Single-cell transcriptome FM trained for drug response modeling | Predicts chemotherapy response in CRC patients from single-cell RNA-seq; enables precision treatment planning | [BoevaLab/CancerFoundation: CancerFoundation: A single-cell RNA sequencing foundation model to decipher drug resistance in cancer](https://github.com/BoevaLab/CancerFoundation) | Pan-cancer foundation model that generalizes well to CRC clinical cohorts. |
